# Supplementary material for: The Female Reproductive Tract Microbiota and Endometrial Cancer: A Systematic Review
Source: Int J Mol Sci. 2025 Sep 12;26(18):8877. doi: 10.3390/ijms26188877 (PMC12470098; doi:10.3390/ijms26188877)
Supplement: Supplementary file 1 [file ijms-26-08877-s001.zip › Supplemental-S1 JBI Analytical Cross-Sectional appraisal matrix .pdf]

Study	Q1 Inclusion criteria clearly defined	Q2 Subjects & setting described in detail	Q3 Exposure measured validly & reliably	Q4 Objective, standard criteria for condition	Q5 Confounders identified	Q6 Strategies to deal with confounders stated	Q7 Outcomes measured validly & reliably	Q8 Appropriate statistical analysis used	Yes count
Walther-António et al., 2016	Yes	Yes	Yes	Yes	Yes	Yes	Yes	Yes	8
Walsh et al., 2019	Yes	Yes	Yes	Yes	Yes	Yes	Yes	Yes	8
Gressel et al., 2021	Yes	Yes	Yes	Yes	Unclear	Yes	Yes	Yes	7
Wang et al., 2022	Yes	Yes	Yes	Yes	Yes	No	Yes	Yes	7
Wang et al., 2024	Yes	Yes	Yes	Yes	Unclear	No	Yes	Yes	6
Leoni et al., 2024	Yes	Yes	Yes	Yes	Yes	No	Yes	Yes	7
Han et al., 2024	Yes	Yes	Yes	Yes	Yes	No	Yes	Yes	7
Ying et al., 2024	Yes	Yes	Yes	Yes	Unclear	No	Yes	Yes	6
Xiao et al., 2024	Yes	Yes	Yes	Yes	Unclear	Yes	Yes	Yes	7
Kaakoush et al., 2022	Yes	Yes	Yes	Yes	Yes	No	Yes	Yes	7
Semertzidou et al., 2024	Yes	Yes	Yes	Yes	Yes	Yes	Yes	Yes	8
Li et al., 2021	Yes	Yes	Yes	Yes	No	No	Yes	Unclear	5
Chen et al., 2024	Yes	Yes	Yes	Yes	Yes	Yes	Yes	Yes	8
Chao et al., 2023	Yes	Yes	Yes	Yes	Yes	No	Yes	Yes	7
González-Bosque et al., 2023	Yes	Yes	Yes	Yes	Yes	Yes	Yes	Yes	8
Barczyński et al., 2023	Yes	Yes	Yes	Yes	Yes	Yes	Yes	Yes	8
Deligdisch et al., 2013	Yes	Yes	Yes	Yes	Unclear	No	Yes	Yes	6
Liu et al., 2023	Yes	Yes	Yes	Yes	No	No	Yes	Unclear	5
Hawkins et al., 2022	Yes	Yes	Yes	Yes	Unclear	No	Yes	Yes	6
Hakimjavadi et al., 2022	Yes	Yes	Yes	Yes	Yes	Yes	Yes	Yes	8
Kuzmycz et al., 2025	Yes	Yes	Yes	Yes	Unclear	No	Yes	Unclear	5

Methodological quality: JBI Analytical Cross-Sectional appraisal matrix
