# Supplementary material for: The Female Reproductive Tract Microbiota and Endometrial Cancer: A Systematic Review
Source: Int J Mol Sci. 2025 Sep 12;26(18):8877. doi: 10.3390/ijms26188877 (PMC12470098; doi:10.3390/ijms26188877)
Supplement: Supplementary file 1 [file ijms-26-08877-s001.zip › Supplemental-S2 methods_summary.pdf]

Study (year)	Bacteria — method/target	Viruses — method	Fungi — method	Platform / key notes
Deligdisch et al., 2013	—	PCR for HMTV	—	Targeted viral detection in endometrial cancer
Walther-António et al., 2016	16S rRNA amplicon (region NR)	—	—	Illumina MiSeq; tract-wide study
Walsh et al., 2019	16S rRNA V3–V5; air monitoring (LB plate)	—	—	Illumina MiSeq; MoBio PowerSoil; confirmatory qPCR
Gressel et al., 2021	16S rRNA V4; QIIME2	—	—	35 hysterectomies; ANCOM/PICRUSt analysis
Li et al., 2021	Integrated microbiome + transcriptome (microbiome source NR)	—	—	Bioinformatic integration; no primary wet-lab
Wang et al., 2022	16S rRNA amplicon (details NR)	—	—	Postmenopausal cohort; tumor/peri-tumor differences
Chao et al., 2022	16S V3–V4 + qPCR validation	—	—	Amplicons + targeted confirmations
Hakimjavadi et al., 2022	Shotgun metagenomics (vaginal microbiome; details/platform NR)	—	—	CSTs and histotypes (LG/HG)
Kaakoush et al., 2022	16S rRNA of 'active microbiota' (transcriptional footprint; details NR)	—	—	Active microbiota and body-weight links
Hawkins et al., 2022	16S rRNA V1–V3	—	—	Methodological guidance and sampling considerations
González-Bosque et al., 2023	Re-analysis of RNA-seq (unmapped reads)	—	—	Computational pipeline (e.g., Kraken/PathSeq; details NR)
Barczyński et al., 2023	Review/synthesis (no primary method)	—	—	Review; no original experimental protocol
Wang et al., 2024	Species-specific qPCR (9 Peptostreptococcus targets)	—	—	Targeted 16S-qPCR; no virome/mycobiome
Leoni et al., 2024	16S profiling/quantification (details NR)	—	—	Intrauterine sampling bias assessment
Han et al., 2024	16S rRNA V3–V4	—	ITS1 (mycobiome)	Illumina MiSeq; standard V3–V4/ITS1 primers
Ying et al., 2024	High-throughput 16S rDNA (region NR)	—	—	Bacterial profiling of endouterine fluids
Xiao et al., 2024	High-depth 16S amplicon	—	—	Intratumoral microbiota sequencing
Chen et al., 2024	Meta-transcriptomics (meta-RNA) on public RNA-seq	Meta-RNA	Meta-RNA	Pipeline for 'active' bacteria/viruses/fungi from RNA-seq
Semertzidou et al., 2024	Method reported (details NR)	—	—	Multi-site; EC with Lactobacillus depletion/high diversity
Kuźmycz et al., 2025	16S V3–V4; QIIME2/DADA2	—	—	Illumina MiSeq; enzymatic lysis; α/β-diversity
